# Multifaceted Roles of DNA Methylation in Neoplastic Transformation, from Tumor Suppressors to EMT and Metastasis

**DOI:** 10.3390/genes11080922

**Published:** 2020-08-12

**Authors:** Laura Casalino, Pasquale Verde

**Affiliations:** Institute of Genetics and Biophysics “Adriano Buzzati Traverso”, CNR, 80100 Naples, Italy

**Keywords:** DNA methylation, neoplastic transformation, tumor suppressors, biomarkers, epithelial-to-mesenchymal transition, metastasis, cancer stem cells

## Abstract

Among the major mechanisms involved in tumorigenesis, DNA methylation is an important epigenetic modification impacting both genomic stability and gene expression. Methylation of promoter-proximal CpG islands (CGIs) and transcriptional silencing of tumor suppressors represent the best characterized epigenetic changes in neoplastic cells. The global cancer-associated effects of DNA hypomethylation influence chromatin architecture and reactivation of repetitive elements. Moreover, recent analyses of cancer cell methylomes highlight the role of the DNA hypomethylation of super-enhancer regions critically controlling the expression of key oncogenic players. We will first summarize some basic aspects of DNA methylation in tumorigenesis, along with the role of dysregulated DNA methyltransferases and TET (Ten-Eleven Translocation)-family methylcytosine dioxygenases. We will then examine the potential contribution of epimutations to causality and heritability of cancer. By reviewing some representative genes subjected to hypermethylation-mediated silencing, we will survey their oncosuppressor functions and roles as biomarkers in various types of cancer. Epithelial-to-mesenchymal transition (EMT) and the gain of stem-like properties are critically involved in cancer cell dissemination, metastasis, and therapeutic resistance. However, the driver vs passenger roles of epigenetic changes, such as DNA methylation in EMT, are still poorly understood. Therefore, we will focus our attention on several aspects of DNA methylation in control of EMT and metastasis suppressors, including both protein-coding and noncoding genes.

## 1. Introduction

During multistep tumor progression, both mutational and nonmutational genomic changes result in the selection and expansion of cancer cell subclones exhibiting selective advantage in the tumor microenvironment (TME), in both primary and secondary sites of cancer growth [1]. In addition to genetic alterations (missense mutations, insertions, deletions, amplifications, translocations, etc.), deranged epigenetic regulation is central to tumor development and progression. Abnormalities in chromatin epigenetic marks include biochemical changes into the DNA backbone, histone modifications, nucleosome rearrangements, and expression of noncoding RNAs. These modifications cooperatively control the gene expression reprogramming in neoplastic cells, along with the multiple cell types recruited to the TME [2].

Pioneering studies on cancer-associated epigenetic reprogramming were mainly focused on DNA methylation, while the interactions between DNA methylation, repressive histone modifications, recruitment of epigenetic readers and nucleosome remodeling represent relatively recent areas of investigation [2,3].

Herein, we will first give an overview of alterations of DNA methylation in cancer cells and then focus on some recent findings on the role of DNA methylation in EMT and metastasis mechanisms.

## 2. Global DNA Hypomethylation and Site-Specific CpG Promoter Hypermethylation Are Hallmarks of Human Cancer

Aberrant DNA methylation is the prevalent epigenetic dysregulation in cancer and consists of both losses (DNA hypomethylation) and gains (DNA hypermethylation) of 5-methyl-cytosine within the CpG dinucleotides. Methylome analyses have shown that, compared to the surrounding healthy tissues, nearly every cancer type at advanced stages holds large hypomethylated regions [4], along with the DNA hypermethylation detectable at distinct loci.

This general conclusion mainly results from comparisons between heterogenous tissue specimens (tumor vs surrounding mucosa), rather than specific cell types. However, multiple lines of evidence show that DNA hypomethylation further correlates with the cancer-cell-type specificity and tumor progression stages. For example, recent works based on the murine *Apc*^min^ model of intestinal tumorigenesis, indicate that DMRs (differentially methylated regions) contribute to the distinction between the early epigenetic alterations occurring in adenomas, compared to the late events promoting tumor progression. These analyses reveal a DNA methylation signature partly conserved in human colorectal cancer [5]. Remarkably, the same model system also highlights the role of the altered DNA methylation in mediating the neoplastic phenotype consequent to the *Apc* functional loss [6].

**Figure 1:**
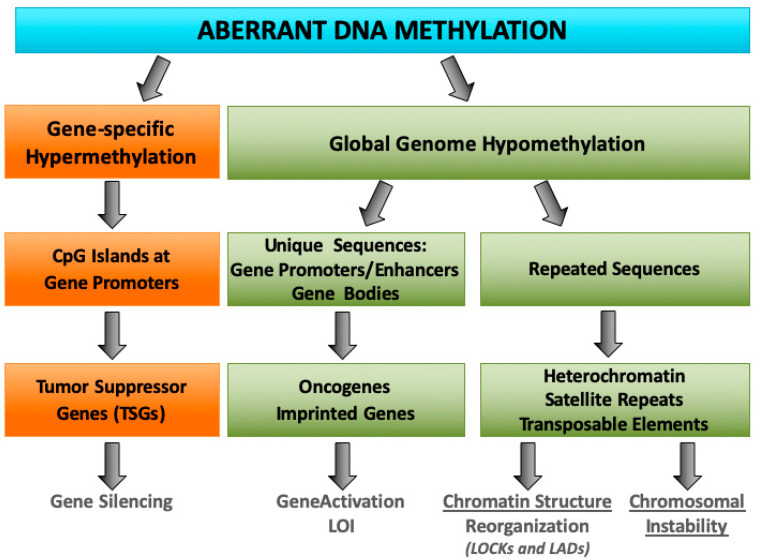
Scheme of aberrant DNA methylation in cancer cells. **Left** (orange): Locus-specific hypermethylation of CpG island in promoter sequences leads to transcriptional inactivation of tumor suppressor genes in cancer cells. **Right** (green): Global cancer-associated hypomethylation affects both unique and repeated sequences. Hypomethylation of unique sequences participates to the activation of oncogenes, mediated by transcriptional enhancers and LOI of imprinted genes involved in cell growth control and tumorigenesis. Hypomethylation of tandem repeats (centromeric and juxta-centromeric satellite DNA), interspersed repeats (Alu and LINE-1), and transposable elements is mainly responsible for chromosomal instability and genomic rearrangements. Loss of DNA methylation within heterochromatic regions corresponding to the LOCKs and LADs (exhibiting high DNA methylation levels and association with nuclear membrane in nonneoplastic cells), results in structural reorganization of large heterochromatin blocks and disorganization of the nuclear membrane.

As summarized in Figure 1, global decrease in DNA methylation and site-specific DNA hypermethylation of promoter-associated CGIs represent common features of the cancer-associated epigenetic landscape. While the genome-wide DNA hypomethylation is associated with oncogene activation and chromosomal instability, the CGIs hypermethylation is linked with repressive chromatin modifications and silencing of tumor suppressor genes (TSG) [7].

The hypomethylated CpGs, which affect both transcription and genome stability, are concentrated in very large hypomethylated domains spreading across vast regions of the cancer cell genome. Hypomethylated blocks largely overlap with LOCKs (large organized chromatin K-modifications marked by H3K27me3 and H3K9me2/me3) and LADs (lamina-associated domains) [8], which represent genomic regions packaged into repressive chromatin structures extensively lost in cancer cells [9]. DNA hypomethylation mainly occurs in regions depleted of genes and enriched for cancer-specific histone-lysine *N*-methyltransferase EZH2 (enhancer of zest homologue 2) binding and repressive marks. Gene-poor domains encompassing constitutively repressed regions are recruited at the nuclear periphery trough lamina association. The repatterning of DNA methylation and heterochromatin structures (loss of histone methylation within LADs and LOCKs) is integrated with an extensive reorganization of the nuclear architecture, with an impact on cancer-associated gene expression [10].

DNA hypomethylation contributes to genomic instability through mechanisms reflecting a whole reorganization of the global genome architecture. In cancer cells, DNA hypomethylation corresponds to hotspots of chromosomal breaks and largely overlaps with repetitive sequences localized within centromeric, pericentromeric, and subtelomeric chromosomal regions, thus resulting in the reactivation of repeat elements. Reactivation of centromeric satellite repeats (microsatellites) and dormant repeat elements, such as the long interspersed nuclear elements (LINEs) is a major determinant of chromosomal instability. Moreover, DNA hypomethylation of LINEs and other transposable elements increases the odds of transposition to other genetic loci, thus contributing to genomic rearrangements by retrotransposition [11].

In addition to the well-established effects of large-scale DNA hypomethylation on genome architecture and chromosomal instability, recent analyses point to the specific function of DNA hypomethylation of distal regulatory sites as a determinant of cancer gene dysregulation [12]. Genome-wide methylome analyses in a large number of normal and neoplastic cell types reveal that, in addition to the profile of histone modifications, transcription factors binding and chromatin looping, pairing between enhancers, and cognate genes can be inferred from DNA methylation data. Importantly, the hypomethylation of a large number of enhancers is associated with overexpression of key subsets (based on gene ontology) of cancer-related genes [12].

Recent genome-wide analyses (bisulfite sequencing of 13 human samples and further validation on almost 700 samples from TCGA (the Cancer Genome Atlas) have been focused on super-enhancer regions, defined as clusters of transcriptional enhancers exhibiting maximal levels of histone H3K27 acetylation and BRD4 recruitment [13]. Most super-enhancers, including the cancer-associated super-enhancers controlling the expression of key oncogenic drivers [14], exhibit altered DNA methylation profiles associated with transcriptional silencing or overexpression of corresponding genes [15] in both solid and hematopoietic cancers [16].

The causal links between cancer-associated enhancer hypomethylation are supported by analysis of enhancer-associated DMRs in various tumors. In hepatic carcinogenesis, among 369 differentially methylated enhancers, a key role is played by the recurrently hypomethylated enhancer of *CEBPB* (encoding for C/EBPbeta, CCAAT/enhancer-binding protein beta), representing a major driver of global transcriptional reprogramming in Hepatocellular carcinoma (HCC) tumor progression [17]. 

Hypermethylation of promoter-associated CGIs likely represents the best studied epigenetic change in tumorigenesis. CGIs are genomic regions at least 200 bp long, with 50% GC dinucleotides and an observed-to-expected CpG ratio > 0.6. CGIs are flanked on both sides by (approx. 2kb-long) CGI shores, regions of DNA with a low density of CpG dinucleotides, where most of the cancer-associated methylation differences are detectable [8].

Hypermethylation of promoter-associated CGIs has been predominantly associated with the transcriptional silencing of TSGs or mismatch repair genes involved in most cancer-relevant pathways [18]. DNA methylation of CGIs results in transcriptional repression mainly by indirect mechanisms, mediated by the recruitment of chromatin silencing factors. However, DNA methylation can also affect transcriptional regulation in a direct fashion by inhibiting (or facilitating) the activity of enhancer regions, which include TFBS (transcription factors binding sites) containing methylated CpG dinucleotides. Methylation triggers the binding of methylated DNA-specific binding proteins to CpG sites, attracting histone-modifying enzymes that, in turn, focally establish a silenced chromatin state [19].

Cancers can be classified according to their degree of methylation, and the tumors with high degrees of methylation represent a clinically and etiologically distinct group characterized by epigenetic instability. Specifically, in some tumors the concurrent multiple promoter hypermethylation of tumor-related genes across the genome is referred to as CIMP (CGI methylator phenotype) [20]. Remarkably, CIMP-associated cancers seem to have a distinct epidemiology, a distinct histology, distinct precursor lesions, and distinct molecular features [21].

## 3. DNA Methyltransferases in Malignant Transformation

The cancer-associated epigenomic reprogramming, involving global DNA hypomethylation and reorganization of heterochromatic regions and hypermethylation of promoter-associated CGIs and CIMP, results from somatic genetic mutations and/or expression changes of various epigenetic regulators [22]. Among DNA methylation modifiers, such as DNA methyltransferases (DNMTs) [23], DNMT1 preferentially methylates hemimethylated CpG sites and maintains methylation following cell division, while DNMT3A and DNMT3B, which recognize both unmethylated and hemimethylated sites, are responsible for de novo methylation at unmethylated CpG sites. Given their functions in both the establishment and maintenance of genomic DNA methylation [24,25] DNMT3A and DNMT3B critically contribute to the aberrant cancer-associated methylation patterns.

5-hydroxymethylcytosine (5-hmC) is a recently identified cytosine modification, resulting from the oxidation of 5-mC, catalyzed by the TET family of methylcytosine dioxygenases (TET1, TET2, and TET3). TET enzymes are responsible for the successive oxidation of 5-mC to 5hmC, 5fC (5-formylcytosine), and 5caC (5-carboxylcytosine). These residues are the intermediates in the demethylation process in which the TDG (thymine DNA glycosylase), by hydrolyzing the bond between the base and deoxyribose ring, produces an AP (A-Pyrimidinic) site that is replaced by an unmethylated cytosine by BER (base-excision repair) [26,27].

Mutations of DNMTs and the resulting dysregulation of genomic methylation are involved in neoplastic transformation and tumor progression. DNMTs abnormalities are associated with a large variety of tumors in which they cause aberrant patterns of DNA methylation [28]. Common lesions affecting the DNMTs genes include overexpression, mutation, and deletion.

In agreement with the DNMT3A role in hematopoietic stem cell differentiation [29], DNMT3A mutations are prevalent in lymphoid [30] and myeloid malignancies [31,32].

In a variety of tumors (esophageal squamous cell carcinoma, HCC, sporadic breast tumors, and colon microadenomas) overexpression rather than mutation of DNMTs (DNMT1, DNMT3A, and DNMT3B) results in hypermethylation and oncogenic activation. Heterogeneous degrees of DNMT1 overexpression are associated with primary colon cancer cells, while DNMT3B overexpression has been related to CIMP-high colon cancer, although the overexpression of the protein not always parallels the RNA levels [21].

More recently, the role of TET proteins in neoplastic transformation has been established by the identification of inactivating mutations, mainly affecting TET2, in various types of cancer with a higher prevalence of hematological malignancies with respect to solid tumors [26,27]. Moreover, mutations of the *IDH1/2* genes, encoding the isocitric dehydrogenases metabolic enzymes, result in the accumulation of 2-hydroxyglutarate, which inhibits the TET protein enzyme activity. The evidence that in some tumors, such as AML (acute myeloid leukemia) *TET2* and *IDH* mutations are mutually exclusive, support their involvement in the same pathway. Accordingly, the TET2- and IDH-mutated clinical samples exhibit similar DNA methylation profiles [33,34].

Despite the number of mechanistic aspects of tumor-associated DNA demethylation that deserve further elucidation, data from model organisms supports the oncogenic function of the loss of DNA methylation. A seminal evidence has been provided by mouse models in which a strong hypomorphic allele or deletion of *Dnmt1* causes DNA hypomethylation and genomic instability leading to aggressive T-cell-induced tumorigenesis, associated with increased mutation rates and aneuploidies [35]. Functional studies in mouse models of colon and prostate tumorigenesis have suggested a tumor suppressor function for *Dnmt1*, whose reduction resulted in increased tumor incidence. In addition, the lack of de novo methyltransferase activity accelerates oncogene-driven carcinogenesis, indicating that also *Dnmt3a* and *Dnmt3b* may act as oncosuppressor genes [36].

Nevertheless, the role of maintenance methylation in tumor development is controversial, since *Dnmt1* can exert oncosuppressor or tumor promoter activity depending on the cancer cell context. In murine models, the MYC transgene induces T-cell lymphomagenesis, while the MLL-AF9 (mixed lineage leukemia-ALL1-fused gene from chromosome 9) chimeric oncoprotein drives AML. Loss of *Dnmt1* delays lymphomagenesis and leukemogenesis by suppressing normal hematopoiesis and impairing tumor cell proliferation and leukemic stem cell self-renewal. Therefore, in this context, DNMT1 may be important for tumor maintenance [36,37]. The increased DNMT1 expression, observed in subsets of human T-cell, B-cell, and myeloid malignancies, further supports this role. Moreover, although somatic mutations of *DNMTs* genes have been described in many tumors and related to aggressiveness and therapeutic resistance, the frequency of *DNMT1* somatic mutations is relatively low (about 3% of colorectal adenocarcinoma and 1.6% of prostate cancer as well as a small subset of cases of AML) [38,39,40].

## 4. The Role of Epimutations in Tumorigenesis and Heritability of Cancer

Epigenetic aberrations represent an emerging mechanism that plays a pivotal role in carcinogenesis. The term epimutation [41] describes the altered epigenetic marks that, similar to genetic mutations, result in transcriptional silencing of active genes or activation of silent genes, thus affecting every stage of tumorigenesis. 

Epimutations include primary epimutations (such as promoter methylation) where no DNA alterations are detected, and secondary epimutations, occurring in concert with (and caused by) a local *cis*-acting DNA alterations [42]. Inheritable epimutations occur at the parental germline level and are often linked to *cis*-acting genetic mutations. Epimutations due to germline variants are widely distributed in all normal somatic tissues. Constitutional epimutations are epigenetic abnormalities arising in early stages of embryonic development and result in a mosaic of both mitotically and meiotically heritable changes. Somatic (de novo) epimutations accumulate in somatic cells in response to environmental stresses and/or ageing. In cancer-affected individuals, epigenetic abnormalities can be selectively found in tumor cells or detected in preneoplastic lesions as well in macroscopically normal tissues adjacent to or within the same organ as the tumor site. The detection of cancer-related somatic epitypes in normal tissues strongly suggests that some epigenetic alterations are acquired prior to carcinogenesis. Somatic epitypes have also been observed in cancer-free individuals and associated with ageing [42]. Environmental factors, including DNA damaging agents, such as ROS (reactive oxygen species), are implicated in these mechanisms. In particular, oxidative damage can target various complexes containing DNA methyltransferases along with the histone deacetylase SIRT1 and Polycomb members (histone methyltransferases) to the promoter-associated CGIs [43].

Somatic epimutations can participate to cancer initiation or contribute to cancer progression by activating oncogenes and prometastatic genes and/or by inactivating growth-inhibitory genes or key TSGs. Silencing of TSGs by de novo aberrant promoter DNA hypermethylation is an early oncogenic event that contributes to clonal expansion of the preneoplastic cell populations and precedes the onset of malignant growth. Epigenetic abnormalities can also represent secondary consequence of neoplastic transformation due to somatic genetic mutations hitting some epigenetic regulator(s). Germline and constitutional epimutations can precede and predispose to cancer development and/or represent a tumor-initiating event. Therefore, constitutional and germline epimutations provide an alternative mechanism to genetic mutation for cancer predisposition and inheritability, thus contributing to the individual cancer susceptibility. However, evidence linking epimutations to cancer risk has been reported for a limited number of genes [42].

Observational studies in cancer-affected families point to the possible role of intergenerational inheritability of constitutional epimutation in human tumorigenesis. Loss of imprinting (LOI) in Wilms’ tumors represents a seminal example of constitutional epimutation. LOI refers to the altered methylation states leading to either biallelic expression or complete silencing of the genes that are normally expressed monoallelically in a parent-of-origin-specific manner. A paradigmatic example is the dysregulated *H19-IGF2* genomic imprinting in Wilms’ tumor. Abnormal methylation of the imprinting control region (ICR) on the maternal allele leads to LOI of the *H19-ICR* locus. The consequently reduced expression of the maternally expressed noncoding transcript *H19* allows the activation of *IGF2*. Therefore, *H19* works as a tumor suppressor, by preventing the biallelic expression of *IGF2* [44].

## 5. DNA Methylation in the Control of Tumor Suppressor Genes

The tumor suppressor genes silenced by aberrant promoter hypermethylation encode a wide range of protein products, including cell cycle inhibitors (INK4A/p16 and Rb), DNA repair factors, detoxifying enzymes (GSTP1, glutathione S-transferase Pi 1), angiogenesis inhibitors (VHL, Von Hippel–Lindau tumor suppressor and THBS1, thrombospondin 1), cell–cell adhesion receptors (CDH1, cadherin-1), metalloprotease inhibitors (TIMP3, tissue inhibitor of metalloproteinases 3), and many others [45,46]. Moreover, in addition to protein-coding genes, DNA hypermethylation is critically implicated in transcriptional downregulation of noncoding RNAs, such as miRNAs (see below).

In agreement with the Knudson two-hit hypothesis [47], hypermethylation of promoter regions often represents the second hit, responsible for the loss of the second allele and consequent inactivation of a wide range of TSGs. A prototype example is represented by the promoter hypermethylation of the *CDKN2A* (cyclin-dependent kinase inhibitor 2A) gene, encoding the INK4A/p16 cell cycle inhibitor, blocking the CDK4/6 activity. *CDKN2A* promoter hypermethylation is responsible for the inactivation of the wild-type allele in CRC cells in which the other allele has been lost by deletion. *INK4A* methylation and loss of p16 expression is an early event in breast and lung cancer (NSCLC, non-small-cell lung carcinoma). Accordingly, in breast cancer specimens, *INK4a* promoter hypermethylation is detectable even in histologically normal human mammary epithelia [48]. Inactivation of the second allele of the tumor suppressor gene *RB1* in human retinoblastoma represents another key example of promoter hypermethylation as the second hit. Similar mechanisms affect various TSGs in tumors associated with familial cancer syndromes caused by heterozygous germline mutations [49].

The major TSGs implicated in DNA repair mechanisms include *MLH1* (mutL homolog-1), *MSH2* (mutS homolog 2), *MGMT* (O6-methylguanine-DNA methyltransferase), and *BRCA1/2* (breast cancer gene 1 and 2) [18].

*MLH1* promoter hypermethylation and epigenetic silencing is implicated in different types of cancers characterized by mismatch repair (MMR) deficiency, which causes insertions or deletions in repeated sequences. Microsatellite instability (MSI) is detected in 90% of hereditary form and 10–15% of sporadic CRCs (colorectal cancers). HNPCC (hereditary nonpolyposis colorectal cancer) or Lynch syndrome is mainly due to germline pathogenic variant in *MLH1* or *MSH2* mismatch-repair genes [50], except for a subset of patients in which CRC predisposition is transmitted by constitutional epimutations affecting the promoter of *MLH1* [51]. Nevertheless, compared to germline genetic mutations, low-level constitutional *MLH1* methylation occurs quite rarely in hereditary forms (Lynch syndrome) and cancer risk associated with the epigenetic mosaicism needs to be ascertained. In contrast, *MLH1* hypermethylation is highly frequent in sporadic CRC with MSI. Therefore, methylated *MLH1* can represent a specific marker for sporadic MSI tumors, which can be used to select patients for genetic testing for Lynch syndrome [51].

While methylation of the promoter region of the *MSH2* mismatch-repair gene is very frequent and associated with relapse in multiple cancers, constitutional *MSH2* epimutation in families with Lynch syndrome is a rare event [52]. Remarkably, *MSH2* methylation in colon mucosa and stemming CRC cells has been found associated with a deletion in an upstream gene (encoding EpCAM, epithelial cellular adhesion molecule) [53].

Similarly, in hereditary CRC no germline or constitutional epimutations have been detected so far in the *MGMT* gene, encoding the O-6-methylguanine-DNA methyltransferase, essential for reversing the addition of alkyl groups to guanine residues. Therefore, its involvement in CRC predisposition remains unexplored and warrants further research [22,54]. In some patients, *MGMT* promoter methylation is found associated with a SNP localized in the first exon [55].

BRCA1, responsible for the chromosomal repair of double-strand breaks, is critically involved in hereditary breast cancer. *BRCA1* promoter methylation has been reported in 30–35% of all triple-negative breast cancers with germline *BRCA1/2* wild-type status, mainly the basal-like subtype. *BRCA1* methylation in endoderm- and mesoderm-derived normal tissues both in patients and in cancer-free individuals supports the incidence of constitutional epimutations (reviewed in [22]). In some cases, epigenetic silencing of *BRCA1* is found associated with a dominantly inherited 5’-UTR variant [56]. 

Genome-wide DNA sequencing and methylome analyses made available by international consortia (such as the TCGA project), confirm the much higher frequency of tumor-associated epigenetic changes compared to genetic mutations. The number of genes silenced by cancer-associated promoter hypermethylation has dramatically increased during the years, reaching almost 10% of the CGI-containing promoters. Therefore, genome-wide CGIs methylation profiling, in addition to providing novel biomarkers for diagnosis and prognostic predictions, is a valuable tool for the identification of novel oncosuppressor genes. For example, analyses of *MLH1* and *CDKN2A*(p16) methylation combined with the CIMP status exhibit clinical and prognostic value in colorectal cancer [57].

In addition to CRC, *MLH1* promoter methylation is a well-established biomarker for multiple solid tumors, including esophageal, NSCL, gastric, bladder, and papillary thyroid cancer. In CRC, methylated *MLH1* is one of the major epigenetic biomarkers along with other genes involved in the tumor progression and metastasis, such as *CDKN2A*/p16(INK4A), *CDKN2A*/p14(ARF), *MGMT*, *TIMP, THBS1 3*, and *THSD1* (thrombospondin type 1 domain containing 1), identified in genome-wide screening for methylation-silenced genes, along with meta-analysis of clinical data. *CDKN2A* hypermethylation correlates with tumor progression, metastasis, and overall survival, thus representing a promising diagnostic and prognostic biomarker in HNSCC (head and neck squamous cell carcinoma) [58].

In addition to the well-known tumor suppressors, novel genes have emerged as candidate TSGs and novel biomarkers. For example, aberrant methylation and silencing of neuropeptides and GPCRs, including galanin and galanin receptors *(GALR1* and *GALR2*) [59], tachykinin-1 and tachykinin receptor (*TACR1*) [60], and somatostatin and somatostatin receptor (*SST* and *SSTR1*) [61] are common in HNSCC. *SST* hypermethylation is also detectable in esophageal, gastric, colon, and renal cancer in which the prognostic power of *GALR1* and *GALR2* epigenetic modifications is further confirmed [62]. Among the tumor-specific epigenetic signatures, analyses of four methylation-silenced genes in a large series of patients of laryngeal and hypopharyngeal cancer shows that hypermethylation in promoter regions of *MGMT* (90%), *DAPK* (death-associated protein kinase) (91%) and *CDH1* (E-cadherin) (81%) (but not p16) is a frequent event, although not predictive of mortality or second primary cancer [63].

The prognostic relevance of the above-mentioned epigenetic biomarkers is further highlighted by their predictive role in the responses to chemotherapeutic treatments. *MLH1* hypermethylation confers resistance to multiple chemotherapeutic drugs in ovarian and colorectal cancer. In human gliomas, *MGMT* downregulation correlates with poor prognosis but also with drug-responsiveness. Accordingly *MGMT* promoter methylation is associated with sensitivity to alkylating agents (temozolomide) [64]. Similarly, the methylation-mediated silencing of *BRCA* genes predicts sensitivity to PARP inhibitors, which are highly effective in the treatment of *BRCA*-positive breast and ovarian cancers (reviewed in [65]).

Recent strategies rely on the genome-wide methylation profiling aimed at the identification of novel prognostic signatures. This is crucial for discriminating the prospectively localized from the tumors at high risk for metastatic progression.

Representative examples include breast and prostate cancer. Genome-wide DNA methylation profiles have been generated from almost 300 tumor tissue specimens and validated in independent datasets and in large numbers of samples from TCGA. These studies have provided a prognostic signature based on methylation changes of 15 CGIs, which correlates with survival of patients diagnosed with invasive breast tumors or DCIS (ductal carcinoma in situ) [66]. Similarly, in prostate cancer, eight differentially methylated CGIs, which allow to distinguish the metastatic-lethal from nonrecurrent tumors, have been identified by methylome analysis of surgical specimens from large cohorts of prostate cancer patients followed up for at least 5 years [67].

Among the innovative protocols aimed at minimally invasive cancer diagnosis, the immunoprecipitation-based cfMeDIP-seq (cell-free methylated DNA immuno-precipitation sequencing) allows to perform cost-effective methylome analyses on small amounts of circulating cfDNA (cell-free DNA). Thus, the large-scale DNA methylation changes allow the detection and classification of several tumor types in patients with early-stage disease [68].

## 6. DNA Methylation in EMT and Metastasis Mechanisms

**Figure 2:**
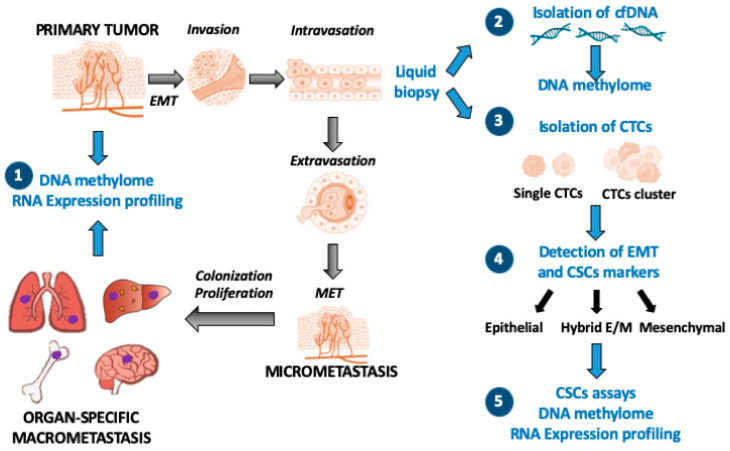
Scheme of the sequential steps of cancer cell dissemination from primary tumor to distant metastases. EMT indicates the Epithelial-to-Mesenchymal Transition required for the gain of invasive properties and anoikis resistance. MET (Mesenchymal-to-Epithelial Transition) refers to the reversion to the epithelial phenotype at sites of metastatic tumor growth. The numbers indicate various strategies for studying the changes of DNA methylation in the multistep metastatic process. (1) DNA methylome and RNA expression profiling in metastatic tissues vs primary tumor specimens; (2) liquid biopsy: DNA methylome analysis in circulating tumor DNA (in cfDNA extracted from blood samples); (3) liquid biopsy: enrichment by microfluidics devices of (single vs clustered) CTCs from blood samples; (4) detection of EMT and cancer-stem-cell markers, by FACS analyses and cell sorting; (5) DNA methylome and RNA expression profiling in various CTC subpopulations (cancer-stem-cell assays: in vitro colonies and spheroids formation).

Metastatic colonization is a complex process, resulting from multiple sequential steps: cells detachment from primary tumor, tissue invasion, intravasation, survival in blood or lymphatic stream, extravasation, and formation of micro-metastases that eventually progress to clinically apparent metastases (Figure 2).

According to the current model of parallel progression of primary tumors and metastasis [69], based on the original Stephen Paget’s “seed and soil” hypothesis [70], metastasis arises from early disseminated tumor cells, which undergo a relatively inefficient transition from micrometastasis to clinically evident lesions. Tumor dissemination is characterized by highly dynamic changes of cancer cell phenotypes during the steps leading to overt metastatic growth. Despite extensive efforts aimed at estimating the genetic heterogeneity between primary tumors and metastases, metastasis-specific driver mutations have been rarely identified. Thus, while genetic alterations are unlikely to play major roles in metastatic transition, the dynamic nature of the epigenetic modifications points to their key contribution to the multistep metastasis process [71,72].

EMT, along with the reverse mechanism (MET, mesenchymal-to-epithelial transition), represents the trans-differentiation process, which allows cancer cells to switch reversibly between epithelial and mesenchymal phenotypes [73]. EMT depends on the reactivation of embryonic transcriptional programs, driven by a complex interplay between EMT-inducing transcription factors (EMT-TFs) and miRNAs [74,75]. Importantly, transition to the mesenchymal phenotype corresponds to the gain of stem-like features, which characterize the therapeutically resistant fraction of tumor-initiating cells (CSCs, cancer stem cells) [76].

Loss of the major component of adherens junctions, E-cadherin (*CDH1*), which is essential for the intercellular contacts supporting epithelial integrity, represents one of the key hallmarks of EMT [77].

One of the early evidences of the *CDH1* CGI hypermethylation was obtained in invasive E-cadherin-negative variants of breast and prostate cancer cell lines [78] and subsequently confirmed in other tumors, including oral [79] and breast [80] cancer. Furthermore, during the metastatic progression of PTC (papillary thyroid cancer) the dynamic changes of *CDH1* epigenetic silencing are functionally related with the in vitro thyroid cancer cell invasiveness the in vivo E-cadherin downregulation in lymph nodal metastases [81].

The well characterized *CDH1* transcriptional repression by various families of EMT-inducing transcription factors (Twist, Snail, ZEB1/2) raises the question on the relationship between EMT transcriptional control and epigenetic modifications at the *CDH1* locus. The time-dependent response of immortalized human mammary cells to sustained EMT-inducing treatments shows that E-cadherin transcriptional repression precedes the methylation of the *CDH1* CGI [82]. The in vitro induced *CDH1* silencing strongly correlates with the promoter methylation of other genes, such as *ESR1* (encoding for the estrogen receptor alpha), silenced in a subset of invasive breast cancer (TNBC, triple-negative breast cancer). Accordingly, both *CDH1* and *ESR1* are represented in a signature of genes silenced by promoter methylation, which characterizes the TNBC, basal-like, and claudin-low breast cancers [83]. These results confirm previous analyses showing that six members of the nine-genes signature are downregulated through promoter methylation in a subset of breast cancer cell lines exhibiting a hyper-methylator phenotype consequent to *DNMT3B* overexpression. The hyper-methylator phenotype, characterizing a distinct cluster of basal-like breast cancers, co-segregates with the worst prognosis due to the high rate of metastases [84]. Moreover, the evidence that selected CGIs controlling specific gene promoters (e.g., *ESR1* and *TWIST1*) are targeted by DNA methylation in response to microenvironmental TGF-beta favors the role of deterministic vs stochastic mechanisms, as responsible for the epigenetic reprogramming in the phenotypically plastic cell populations undergoing EMT [82]. 

Mesenchymal transformation of human breast cancer cells can take place in vivo, without TGF-beta treatment, in a mouse xenograft model of *HRAS*-transformed (MCF10A-derived) cell lines. In this breast cancer progression system, increased activity of the TGF-beta-Smad2 signaling pathway is associated with the DNA methylation-mediated silencing of a gene subset including *CDH1*. Along with the mesenchymal cancer cell phenotype, inhibition of TGF-beta signaling is able to revert the methylation status and expression of select genes by inhibiting the DNMT1 and DNMT3B binding to the *CDH1* and other promoter regions [85].

The global changes of DNA methylome in the TGF-beta induced EMT in ovarian cancer cells show that the promoter methylation and decreased expression of EMT hallmarks, such as *CDH1* and *COL1A1* (Collagen Alpha-1 Chain), are paralleled by the TGF-beta-induced expression and activity of DNMTs (1, 3A and 3B). Accordingly, the treatment with a DNMT inhibitor prevents the TGF-beta-induced EMT [86].

Among the well-characterized EMT-inducing factors, hypoxia, as TGF-beta, results in a global epigenetic reprogramming and changes of the DNA methylome. The hypoxia-induced EMT depends on the demethylation of a subset of genes (with a major role played by *INSIG1,* insulin-induced gene 1) by the TET1 dioxygenase, induced by hypoxia in multiple cancer cell lines. Interestingly, in addition to its role in demethylating 5-mC, TET1 also works as a coactivator of HIF1, participating to the transcriptional induction of the hypoxia-responsive genes [87].

In a mouse cell line, in which EMT is driven by the constitutive expression of a nonhistone chromatin component (HMGI-C, encoded by *HMGA2*), the HMGI-C chromatin remodeling activity contributes to the *Cdh1* hypermethylation by recruiting Dnmt3a on the *Cdh1* promoter, in addition to upregulating the *Dnmt3a* expression [88].

Moreover, E-cadherin silencing is clinically relevant in stomach tumorigenesis in which *CDH1* promoter methylation represents the second hit following the *CDH1* mutation, in both hereditary [89] and sporadic [90] diffuse gastric cancer (GC). The expression of the DNMT3A isoform b (but not DNMT3Aa) correlates with TNM stage and lymph nodal metastasis in GC patients. Mechanistically, DNMT3Ab promotes EMT, associated with DNA hypermethylation and repressive histone modifications (H3K9me2 and H3K27me3) by cooperating with the EMT-TF SNAIL at the *CDH1* promoter in response to TGF-beta. While DNMT3Ab inhibition reduces EMT and metastasis, ectopic DNMT3Ab affects the expression of key metastasis-related genes, encoding for extracellular proteases, fibronectin, and tight junction components. In agreement with similar observations with DNMT3Aa in other tumors, DNMT3Ab impacts the TGF-beta-Smad pathway, and DNMT3Ab inhibition abrogates the TGF-beta-induced EMT [91].

In addition to E-cadherin (*CDH1*), several relevant metastasis suppressor genes are differentially methylated and transcriptionally silenced in metastatic lesions with respect to primary tumors (Table 1).

The nucleoside diphosphate kinase NM23, one of the first metastasis suppressors identified in melanoma and breast cancer, inhibits the in vitro invasiveness of multiple cancer cell types. NM23 in vivo expression inversely correlates with the methylation of one of two promoter-flanking CGIs [92]. NM23 exhibits multiple biochemical activities, including the inhibition of the MEK/ERK signaling pathways, resulting from the NM23-mediated histidine phosphorylation of KSR1 (kinase suppressor of Ras1), one of the scaffold factors of the RAFMEK/ERK cascade [93].

RKIP (Raf kinase inhibitory protein) is another metastasis suppressor impinging on the proinvasive MAPK pathway, by inhibiting the Raf-1 (but not B-Raf) kinase activity [94]. The prognostically significant *RKIP* promoter hypermethylation has been characterized in breast, esophageal, gastric carcinomas, and other cancers in which loss of RKIP expression correlates with poor prognosis. In both in vitro and in vivo breast cancer models, re-expression of RKIP blocks multiple steps of invasion and metastasis through a signaling cascade involving LIN28 and let-7 downstream to the MAPK pathway. Interestingly, RKIP inhibits the invasive potential of breast cancer cells without affecting tumor growth [95].

Among the more recently characterized metastasis suppressors affecting the MAPK pathway, the *SHISA3* gene product inhibits the TRIM21 (tripartite motif containing 21 E3 ubiquitin-proteinigase)-dependent degradation of SGSM1 (small G-protein signaling modulator 1), which negatively controls the MEK/ERK signaling. When *SHISA3* is silenced, the increased SGSM1 polyubiquitylation and degradation abrogates one of the brakes impinging on the MAPK pathway. Accordingly, SHISA3 is downregulated in various tumor types, including nasopharyngeal carcinoma (NPC) in which the *SHISA3* promoter hypermethylation correlates with the SHISA3 ability to suppress the NPC in vitro invasion and in vivo lymph node metastasis [96]. 

The metastasis suppressor *RECK* encodes for a membrane glycoprotein (RECK, reversion-inducing cysteine-rich protein with kazal motifs), which negatively regulates the ECM-degrading metalloproteases. *RECK* is silenced by DNMT3b-mediated promoter methylation in lung cancer cells in which RECK suppresses invasiveness [97] and inversely correlates with lymph node metastasis in NSCLC [98], PDAC (pancreatic ductal adenocarcinoma) [99], osteosarcoma [100], esophageal [101], and breast cancer [102].

Other metastasis-suppressor gene products act as transcriptional regulators. In particular, *BRMS1* (breast cancer metastasis suppressor 1)—which inhibits breast (but also ovarian, melanoma, NSCLC, and bladder) cancer, in addition to cancer metastasis without affecting in vitro and in vivo tumor growth—participates to the mSin3 histone deacetylase transcriptional repressor complex. Interestingly, in addition to correlating with the pathological staging in breast cancer and NSCLC [103,104], the *BRMS1* (breast cancer metastasis suppressor 1) promoter methylation represents a relevant example of prognostic biomarker detectable by liquid biopsies in both cfDNA [104] and CTCs (circulating tumor cells) [105].

The prognostic value of DNA methylation analysis in cfDNA has been recently applied to the prediction of therapeutic resistance in multiple solid tumors. In addition to the ability to disseminate and metastasize, the EMT also confers resistance to both cytotoxic and targeted therapies. Since the DNA methylation-driven mesenchymal transition correlates with therapeutic resistance, monitoring of DNA methylation changes in EMT genes allows to predict the tumor responsiveness and acquired resistance to sorafenib in patients with advanced hepatocellular carcinoma [106]. The role of DNA methylome analyses and liquid biopsies, along with the detection of EMT markers, is summarized in Figure 2.

## 7. DNA Methylation in EMT and Metastasis: Roles of miRNAs

In addition to protein-coding genes, the search for metastasis suppressors has been extended to noncoding RNAs. By correlating the methylation of CGIs and expression profiles of miRNA-containing genes in metastatic cancer cell lines treated with demethylating drugs, several miRNAs reactivated by promoter demethylation were identified and characterized for their in vitro and in vivo anti-invasive and antimetastatic activity [107] (Table 2).

The interplay between the EMT-TFs and miRNAs is a major control mechanism of EMT, invasion and metastasis. The EMT-TFs (E-cadherin repressors) are connected to miRNAs by double-negative feedback loops in which the miRNA targets the EMT-TF, which in turn, transcriptionally inhibits the cognate miRNA. Well-studied examples include the miR-200-ZEB1/2, miR-34a/b/c-SNAIL, miR-15a/16-1-AP4, etc. These regulatory feedback loops are further controlled by the p53 tumor suppressor, which, as transcriptional inducer of the three miRNA families (miR-200, miR-34 and miR-15/16), shifts the balance towards the miRNAs, thus contributing to the maintenance of the epithelial state [75,108].

During tumor progression, promoter hypermethylation is a major mechanism of silencing of the miR-34 family of oncosuppressor miRNAs transcribed from two genetic loci, encoding for miR-34a and miR-34bc [107,109,110,111,112]. In a variety of solid tumors, downregulation of miR-34 family members, in addition to affecting primary tumor growth, is implicated in EMT and metastasis mechanisms and represents a negative prognostic factor. The target transcripts that mediate the metastasis-suppressor activity of miR-34 include, in addition to Snail1 [113], other key regulators of cancer cell invasion and EMT, such as the Notch1 [113], IL6R, and Axl [114] receptors along with the Fra-1 [115,116] and ZNF281 [117] transcription factors.

The miR-200 family members, encoded by two miRNA clusters localized on chromosome 1 (miR-200a, miR-200b and miR-429) and chromosome 12 (miR-200c and miR-141), are highly expressed in cancer cells with epithelial features and downregulated in phenotypically mesenchymal cells, in which they are repressed by the EMT-TFs ZEB1 and ZEB2. Extracellular EMT-inducing cytokines, such as TGF-beta, transcriptionally stimulate *ZEB1*, and/or *ZEB2*, thus interrupting the miR-200-ZEB1/2 negative feedback loop. Interestingly, in an in vitro EMT cell system, prolonged TGF-beta treatment induces the DNA methylation of the five miR-200 promoter regions [118] in agreement with the reports on the DNA hypermethylation of the miR-200 family members in the invasive variants of various tumors [119,120,121]. In invasive breast cancer, hypermethylation and silencing of the miR-200 family members is associated with EMT features, lymph nodal metastasis and loss of ER (estrogen receptor) and PR (progesterone receptor) expression [122,123]. Moreover, in nontumorigenic breast basal cell lines spontaneously undergoing EMT, hypermethylation of the miR-200c-141 locus is paralleled by the upregulation of the EMT-TFs [124]. In summary, in a large variety of cancers, DNA methylation affects the *CDH1* transcription by both direct and indirect mechanisms, mediated by hypermethylation of the *CDH1* promoter and/or DNA hypermethylation-mediated transcriptional downregulation of the miRNAs targeting the E-cadherin repressors (EMT-TFs).

Although E-cadherin represents the best characterized target of ZEB1/2 in EMT, the miR-200-ZEB1/2 axis also controls other key genes, such as *CRB3* and *LGL2*, involved in the control of epithelial cell polarity. The CGI hypermethylation, along with the associated histone modifications of the two loci encoding the five miR-200 family members, is a dynamic process, which can be reversibly triggered by extracellular cues, such as TGF-beta. In addition, the methylation-mediated silencing of miR-200 takes place in experimental models of liver metastasis. Accordingly, in laser micro-dissected human colorectal cancer samples miR-200 CpG hypermethylation and silencing are detectable in mesenchymal cells (belonging to both tumor and normal stroma) [125].

Along with miR-200 and miR-34 family members, silencing of other CpG-hypermethylated miRNAs favors the metastatic process through several proinvasive pathways. In pancreatic cancer, hypermethylation of the miR-124 promoter region (also observed in other tumors) correlates with tumor progression and metastasis, through mechanisms at least partially mediated by miR-124. miR-124 targets the RAS-superfamily GTPase Rac1, a well-characterized driver of cell motility, which in turn, drives cell migration and invasion through the MKK4-JNK-c-Jun pathway in pancreatic cancer cells [126].

DNA methylation is the major control mechanism of genomic imprinting. The 14q32 locus encompasses three paternally expressed protein-coding genes and a large variety of maternally expressed noncoding genes (lncRNAs, snoRNAs, piRNAs), also including a cluster of 54 miRNAs. In CRC cells, both genetic and pharmacological strategies reactivate the 14q32 miRNA cluster. Drug-induced reactivation is mediated by the MEG3 (maternally expressed gene 3)-DMR regulatory element, which controls the transcription of the 14q32 miRNAs, through mechanisms dependent on the recruitment of CTCF (CCCTC-binding factor) to the demethylated MEG3-DMR. Ectopic re-expression of four members of the 14q32 miRNA cluster is sufficient to inhibit liver metastasis by colorectal cancer cells in agreement with clinical data showing the association of increased 14q32 miRNAs expression with limited metastatic spread and better prognosis [127].

It is well established that the EMT generates cells with properties of stem cells [76]. Accordingly, the DNA methylation-dependent epigenetic silencing of the miR-200 family members [124], along with the similarly regulated miR-203 [128] and miR-34c [129], contributes to the gain of stem-like features and self-renewal ability.

## 8. Roles of DNA Methylation in CTCs and CSCs (Cancer Stem Cells)

Besides being a promising new technique for early cancer diagnosis and treatment, noninvasive liquid biopsy has strongly impacted the studies on the role of DNA methylation, EMT, and metastasis. In particular, various methods have been developed to detect and isolate the CTCs from easy-access small blood samples for downstream analysis. Protocols based on physical features (size- and density-based methods), antibody-mediated capturing (immunoaffinity-based), or functional assays (or combinations of these methods) allow real-time information on tumor staging (metastatic vs. nonmetastatic) and the molecular profiling of CTCs.

As expected on the basis of the role played by EMT in intravasation and anoikis-resistance, CTCs from patients affected by the major solid tumors exhibit prevalent expression of mesenchymal vs epithelial markers [130].

CTCs isolated from breast cancer patients include cell populations predominantly expressing mesenchymal markers but also epithelial and E/M subpopulations, coexpressing both epithelial and mesenchymal markers) [131]. The detection of hybrid EMT features in CSCs agrees with the concept that EMT is not an all-or-nothing phenotypic switch but a continuum of intermediate cellular states [74].

The evidence that the mesenchymal CTCs derived from breast tumors are present as both single cells and multicellular clusters [131] agrees with the current models, suggesting that metastatic dissemination requires the formation of multicellular clusters (or CTMs, circulating tumor microemboli), resulting from both homotypic and heterotypic interactions with other cell types (fibroblasts, leukocytes, and platelets) [130]. These observations are supported by single-cell profiling of CTCs, which highlights aspects of tumor heterogeneity not revealed by analyses of circulating cfDNA [132]. The role of DNA methylome analyses in CTCs isolated from liquid biopsies, along with the detection of EMT markers is summarized in Figure 2.

Recently, to understand the role of CTC clusters in metastasis mechanisms, the genome-wide methylation landscape of single CTCs and CTC clusters has been investigated in samples from both breast cancer patients and mouse models. One major difference is represented by the methylation of the genomic binding sites for stemness-associated transcription factors (OCT4, NANOG, SOX2, and SIN3A), which are specifically hypomethylated (while Polycomb target genes are hypermethylated) in CTC clusters compared to single CTCs. Moreover, the clustering of CTCs is a determinant of methylome remodeling and hypomethylation of the stemness TFBSs (coinciding with the activation of respective transcriptional modules). These results were obtained by the treatments with drugs that dissociate the CTCs clusters. Accordingly, the same drugs inhibit the formation of metastasis in mouse models and the DNA hypomethylation profile of CTCs clusters correlates with a subset of breast cancers exhibiting shorter PFS (progression free survival) [133].

The contrast between these findings and the key role of EMT in metastasis is only apparent if considering the lines of evidence showing that the hybrid E/M, rather than the fully mesenchymal phenotype, is essential for maximal tumorigenicity of TNBC cells [134]. Remarkably, the collective mode of invasion by the clusters of CTCs is led by a fraction of stem-like cells with the E/M hybrid features [135]. Along with the epigenome remodeling elicited by changes of intercellular adhesion, the same report [133] points to the relevance of the DNA hypomethylation of transcription factors binding sites in the control of stem-like transcriptional programs in metastasizing cancer cells.

The extensive analysis of eDMRs (enhancer differentially methylated regions) in multiple datasets representing both primary (benign and malignant) tumors shows that most methylation variation occurs at enhancers. eDMRs analysis allows the classification of primary tumors according to the organ system and correlates with the likelihood of metastasis. The eDMRs plasticity is associated with the prognosis of melanoma patients. Interestingly, mimicking the melanoma bone metastasis microenvironment (by coculture with osteoblasts) recapitulates the expression changes of the eDMRs-associated genes altered in bone metastasis [136]. In addition, the chromatin distribution of pluripotency transcription factors, such as *SOX2* and *NANOG*, is higher within eDMRs than within differentially methylated CGIs or promoters in agreement with the findings obtained in CTCs [133].

The EMT and the upregulation of pluripotency transcription factors result in the gain of stem-like features, which characterize the CSC population, responsible for tumor initiation, metastatic dissemination and therapeutic resistance.

Recent findings point to the key role of DNA methylation in the switch between CSCs and non-CSCs. In CSCs, *NANOG* shows ubiquitous expression correlating with the hypomethylation of two of the CGIs associated with the *NANOG* promoter in response to the miR-135a/DNMT1/SMYD4 axis. The switch from non-CSCs to CSCs is associated with miR-135a upregulation. miR-135a, in turn, directly targets *DNMT1*, thus allowing SMYD4 to bind the unmethylated *NANOG* promoter and activate its expression. Moreover, the proinflammatory cytokine TNF-alpha triggers the FOXM1 (Forkhead Box M1)-mediated induction of miR-135a, enabling the transformation of non-CSCs (Nanog−) into CSCs (Nanog+). Therefore, the TNF-alpha/FOXM1/miR-135a/DNMT1/SMYD4 pathway links the control of *NANOG* expression to the inflammatory TME [137].

In contrast with the cancer cell stemness-associated demethylation of the *NANOG* promoter, the silencing of the *H1.0* (encoding the H1.0 linker histone) is required for maintenance of the self-renewing CSCs fraction. According to single-cell gene expression and methylation analysis of the *H1F0* locus in clinical samples, compared to various TCGA datasets, in multiple tumor types H1.0 is heterogeneously expressed in non-CSC tumor cells, while the self-renewing CSC population exhibits the lowest levels of the protein. These changes result from differential methylation of an enhancer region (characterized by high H3K27ac) within a CGI shore, upstream to the *H1F0* promoter. The lack of H1.0 causes the destabilization of nucleosome-DNA interactions and coordinated de-repression of neighboring genes, resulting in the activation of transcriptional networks involved in cancer cell self-renewal [138].

## 9. Concluding Remarks and Future Perspectives

We have summarized several aspects concerning the gene expression changes associated with abnormal DNA methylation in neoplastic transformation with a focus on DNA methylation in EMT and metastasis mechanisms.

DNA methylation represents one of the key epigenetic players, along with the recently emerged noncoding RNAs, including the relatively well-characterized miRNAs. As above described, the EMT-associated gene expression programs are finely tuned by multiple feedback loops involving the major metastasis-suppressor miRNAs and several EMT-TFs.

Similarly, a novel regulatory layer is represented by the mutual regulation of miRNAs and DNA methylation, involving the miRNA-mediated regulation of both *DNMTs* and methyl-CpG binding proteins, which in turn, control the transcription of miRNA-coding genes [139]. These mechanisms deserve future investigations in the framework of studies on DNA methylation in tumor progression and metastasis.

As summarized in this review, DNA methylation critically controls the expression of a wide range of oncosuppressor and metastasis suppressors. Functional validation generally relies on the effect of global gene reactivation by treatment with demethylating agents and/or ectopic overexpression of individual genes. However, several recently developed methods, derived from the CRISPR-Cas9 editing tool and based on the sequence-specific recruitment of the catalytic domain of the TET1 dioxygenase, allow the targeted demethylation at specific sites [140]. This approach has made possible the programmable reactivation of the promoter region of the *BRCA1* tumor suppressor [141]. These strategies open novel perspectives that will allow the fine dissection of epigenetic regulatory pathways and the design of new therapeutic tools aimed at the selective reactivation of individual tumor suppressor genes.

The application of diagnostic and prognostic tools based on DNA methylation analyses depends on the availability of low-cost methods for mapping DNA methylation at single-nucleotide resolution along with genomic datasets, such as the TCGA database, which represents most tumor types in very large cohorts of patients. The integration of genome-wide DNA methylation analyses with expression profiling and clinical data will require increasingly powerful computational algorithms. For example, the recently developed RESET (resource to detect epigenetically silenced and enhanced targets in cancer) has been exploited for obtaining a pan-cancer landscape of aberrant DNA methylation, by integrating the DNA-methylation data and cis-transcriptional changes across 6000 human tumors from 24 cancer types [142]. By identifying the oncogenic pathways affected by epigenetic silencing and enhancing events, these approaches will strongly contribute to be innovative therapeutical strategies. 

Moreover, promising future perspectives include the studies on DNA-methylation in therapeutic resistance. Given the role of EMT in controlling the fraction of therapeutically resistant CSCs, it will be important to define the DNA-methylation changes implicated in the mechanisms linking EMT, CSCs, and therapeutic resistance [143]. Accordingly, recent findings point to the role of DNA methylation-driven EMT as a common mechanism of cross-resistance to both targeted treatments and chemotherapy. The in vitro findings, obtained in various cancer cell types, are confirmed by clinical findings in HCC patients in which the DNA methylation status of CGIs of EMT genes emerges as a predictive marker of resistance to sorafenib [106].

The analyses of tumor-associated DNA methylomes in very low amounts of cfDNA is a key advantage of liquid biopsies. This approach, however, differing from the analysis of DNA from CTCs, does not allow us to correlate the DNA methylation changes with the cancer cell phenotypes. On the other hand, the possibility to investigate the EMT and stem-cell markers in CTCs offers the opportunity to study the in vivo role and prognostic correlations of the DNA methylation changes in the cell fraction implicated in metastatic dissemination [144]. Therefore, the availability of innovative microfluidic devices and methods for the enrichment, identification, and isolation of CTCs [145] will strongly contribute to the in vivo studies on DNA methylation in EMT, the gain of stem-like features, and tumor dissemination.

## Figures and Tables

**Table 1:** Representative EMT and metastasis-suppressor protein-coding genes silenced by DNA methylation.

Protein	Gene	Function	Cancer type	Ref
E-cadherin	*CDH1*	Calcium-dependent adhesion protein (adherens junction). Cell-cell adhesions, motility and proliferation of epithelial cells.	Breast And Prostate	[78]
			Oral	[79]
			Breast	[80,85]
			Basal-like Breast	[84]
			Papillary Thyroid Carcinoma	[81]
TWIST1	*TWIST1*	Basic helix–loop–helix transcription factor binding to E box sequences. Cell lineage determination and differentiation. EMT-TF	Multiple Adenocarcinomas	[82]
NM23	*NME1*	Nucleoside diphosphate kinase multifunctional protein metastasis suppressor gene.	Melanoma and Breast	[92]
RKIP	*RKIP*	Member of phosphatidyl-ethanolamine-binding-protein (PEBP) family. Modulator of intracellular signaling pathways.	Breast, Esophageal, and Gastric Carcinomas	[95]
SHISA3	*SHISA3*	Member of a family of transmembrane adaptors modulating both WNT and FGF signaling. Maturation of presomitic mesoderm cells.	Nasopharyngeal Carcinoma	[96]
RECK	*RECK*	Membrane-anchored cysteine-rich glycoprotein with protease-inhibitor-like domains. Negative regulator for matrix metalloproteinase-9. Tumor invasion inhibition.	Lung Cancer	[97]
			NSCLC	[98]
			PDAC (Pancreas)	[99]
			Osteosarcoma	[100]
			Esophageal	[101]
			Breast	[102]
BRMS1	*BRMS1*	Transcriptional Repressor. Anoikis Regulator. Metastasis Suppressor.	Breast	[103]
			NSCLC	[104]

**Table 2:** Representative EMT and metastasis-suppressor noncoding genes (miRNAs) silenced by DNA methylation.

Family	miRNA	Gene	Functional Target	Cancer Type	Ref
miR-34	miR-34a miR-34b miR-34c	*MIR34A MIR34B/C*	SNAIL	Hematological (leukemias, lymphomas) and solid tumors (breast-, lung-, colon-, kidney-, bladder-, and pancreatic carcinoma)	[107]
					[109]
					[110]
					[111]
			SNAIL/c-Met/β-catenin	CRC (Colorectal)	[112]
			SNAIL/Notch	PDAC (Pancreas)	[113]
			IL6R/Axl	NSCLC, CRC, and Breast	[114]
			Fra-1	CRC (Colorectal)	[115]
				Breast	[116]
			ZNF281	CRC (Colorectal) Breast CSCs	[117,129]
miR-200	miR-200a miR-200b miR-200c miR-141 miR-429	*MIR200A MIR200B MIR200MIR141 MIR429*	ZEB1/ZEB2	Breast	[118]
				Breast, Prostate	[119]
				NSCLC (Lung)	[120]
				Bladder	[121]
				Breast	[122]
				Breast	[123]
				CSCs (Breast)	[124]
miR-124	miR-124-1 miR-124-2 miR124--3	*MIR124-1 MIR124-2 MIR124-3*	Rac1	PDAC (Pancreas)	[126]
miR-203	miR-203a miR-203b	*MIR203a MIR203b*	NEBL, NID1, OLFML3, PPAP2B, TFPI	Breast CSCs	[128]

## Abbreviations

TET—ten-eleven translocation; EMT—epithelial-to-mesenchymal transition; CGI—CpG island; LOCKs—large organized chromatin K-modifications; LADs—lamina-associated domains; LINEs—long interspersed nuclear elements; TCGA—the Cancer Genome Atlas; DMRs—differentially methylated regions; HCC—hepatocellular carcinoma; TFBS—transcription factors binding site; CIMP—CpG island methylator phenotype; DNMT—DNA methyltransferase; AML—acute myeloid leukemia; LOI—loss of imprinting; ICR—imprinting control region; THBS1—thrombospondin 1; CDH1—cadherin 1; NSCLC—non-small-cell lung carcinoma; CDKN2A—cyclin-dependent kinase inhibitor 2A; INK4A/p16—p16 CDK 4 inhibitor; MLH1—mutL homolog 1; MSH2—mutS homolog 2; MGMT—O6 methylguanine DNA methyltransferase; BRCA—breast cancer gene; MSI—microsatellite instability; MMR—mismatch repair; CRC—colorectal cancer; HNSCC—head and neck squamous cell carcinoma; cfDNA—cell-free DNA; ESR1—estrogen receptor alpha; TNBC—triple-negative breast cancer; RKIP—Raf kinase inhibitory protein; SHISA3—Shisa family member 3; NPC—nasopharyngeal carcinoma; RECK—reversion-inducing cysteine-rich protein with kazal motifs; BRMS1—breast cancer metastasis suppressor 1; CTCs—circulating tumor cells; CSCs—cancer stem cells; eDMRs—enhancer differentially methylated regions.
